# Comparative Assessment of APTT Reagents for Evaluating Anticoagulant Sensitivity of Fucosylated Glycosaminoglycans (FGs) Derived from Sea Cucumbers

**DOI:** 10.3390/md21110568

**Published:** 2023-10-29

**Authors:** Huifang Sun, Shasha Yang, Pengfei Li, Xiaolei Shang, Pin Wang, Jiali Zhang, Lin Yuan, Ronghua Yin, Na Gao, Jinhua Zhao

**Affiliations:** 1 School of Chemistry and Materials Science, South-Central University for Nationalities, Wuhan 430074, China; sunhuifang2021@outlook.com; 2 School of Pharmaceutical Sciences, South-Central University for Nationalities, Wuhan 430074, China; yangshachen@outlook.com (S.Y.); 13708322361@163.com (P.L.); 15969709010@163.com (X.S.); wangpin1994@163.com (P.W.); zhangjiali20221123@163.com (J.Z.); yuanlin@scuec.edu.cn (L.Y.); yinrh77@163.com (R.Y.)

**Keywords:** fucosylated glycosaminoglycan, intrinsic Xase inhibitor, APTT, ellagic acid, colloidal silica

## Abstract

Fucosylated glycosaminoglycans (FGs) derived from sea cucumbers exhibit potent intrinsic Xase (iXase) inhibition, anticoagulation, and antithrombosis. Plasma activated partial thromboplastin time (APTT), a widely used screening test worldwide, is crucial for evaluating anticoagulant efficacy. However, the applicability of these commercially available APTT reagents for assessing anticoagulation of FGs remains unreported. In this study, we investigated the disparity between ellagic acid and colloidal silica APTT reagents in evaluating anticoagulation of dHG-5 and dHLFG-4, two depolymerized FGs, and elucidated the underlying rationale. The results demonstrated that dHG-5 and dHLFG-4 exhibited heightened sensitivity to the ellagic acid APTT reagent both in vitro and in vivo, and did not significantly affect the activation of APTT reagents for plasma. In addition, both ellagic acid and colloidal silica APTT reagents inhibited the anti-iXase of dHG-5 and dHLFG-4, and the inhibition of the ellagic acid APTT reagent was less pronounced compared to the colloidal silica APTT reagent. These findings suggest that the reduced impact of the ellagic acid APTT reagent on the anti-iXase activity of dHG-5 and dHLFG-4 is responsible for the increased sensitivity in plasma APTT analysis. This study offers valuable insights into the characteristics of two APTT reagents applied for assessing the anticoagulant activity of FG-related compounds.

## 1. Introduction

Thrombotic diseases exhibit a substantial burden of morbidity and mortality, particularly among patients afflicted with infectious conditions [1,2], malignancies [3,4], and operative interventions [5]. Historically, the majority of patients have been administered heparin or oral warfarin; however, due to their narrow therapeutic index and the necessity for frequent laboratory monitoring, it is recommended to utilize low molecular weight heparin (LMWH), such as enoxaparin, which offers a more predictable pharmacokinetic profile and anticoagulant effect [6]. To address the need for safer anticoagulants, a number of novel agents have been developed, including direct thrombin inhibitors (e.g., dabigatran) and factor Xa inhibitors (e.g., rivaroxaban, apixaban) [6]. Currently, there is a growing anticipation for the development of inhibitors targeting the contact activation pathway (also known as the intrinsic system) within the coagulation cascade to effectively mitigate bleeding rates [7,8,9,10]. Among these inhibitors, iXase inhibitors may exhibit enhanced efficacy and safety because iXase is the final and rate-limiting enzyme in the contact activation pathway [11].

Native FGs derived from sea cucumbers, consisting of a chondroitin sulfate-like backbone and unique sulfated fucose-containing branches, have displayed remarkable anticoagulant and anti-thrombotic activities, but also have side effects such as factor XII activation and platelet aggregation [12]. Therefore, depolymerized FGs have been obtained to eliminate these side effects while maintaining anticoagulant and anti-thrombotic activities, benefitting from the development of chemical depolymerization methods, such as free radical depolymerization, nitrous acid deaminative and β-eliminative depolymerization [13,14,15,16]. Significantly, based on the detailed structure–activity studies of FG-related derivatives, dHG-5 as a potent inhibitor of iXase is being developed as a novel anticoagulant drug, with no adverse effects on platelet aggregation and factor XII. It is produced according to β-eliminative procedures from a highly regular natural FG extracted from the sea cucumber *Holothuria fuscopunctata*, with an average molecular weight (Mw) of about 5.2 kDa [17]. dHG-5 is a multicomponent entity consisting of a series of oligosaccharides with different degrees of polymerization, whose respective structures, contents, and contributions to pharmacological activities have been extensively elucidated [18]. Currently, it has received approval from the US Food and Drug Administration and the National Medical Products Administration (NMPA) of China to commence Phase I clinical trials, indicating its potential as a breakthrough anticoagulant drug targeting iXase and offering new perspectives in the field of marine drug development.

APTT analysis is a cost-effective and easily automated coagulation screening test. It reflects the comprehensive activity of coagulation factors such as VIII, IX, XI, II, and fibrinogen, which belong to the contact activation pathway (XI, IX, and VIII) and common (II and fibrinogen) pathway of blood coagulation [19]. In addition to screening and identifying coagulation factor deficiencies, diagnosing bleeding disorders, and monitoring heparin anticoagulant therapy, APTT analysis is also used to evaluate the anticoagulant efficacy of intrinsic coagulant factor inhibitors [20,21]. The APTT test, being a valuable and relatively simple diagnostic tool, has led to the development of various commercially available APTT reagents [22]. These APTT reagents consist of surface activators and phospholipids, and the surface activators mainly include two types of substances: silicon (e.g., colloidal silica, microsilica, and kaolin) and ellagic acid [14,23,24]. The differences in sensitivity of these commercial APTT reagents in some applications have been studied for more scientific use, such as in the detection of mild coagulopathies and lupus anticoagulant [25,26]. 

In the process of developing new drugs, the evaluation of efficacy through appropriate assay systems is of great importance. As part of routine laboratory screening tests conducted worldwide, the suitability of various APTT reagents for different anticoagulants, including heparin, dabigatran, and rivaroxaban, has been assessed [27,28,29,30]. However, the applicability of these APTT reagents in assessing the anticoagulant effect of FG compounds has not been reported. Recently, for the purpose of choosing suitable APTT reagents used in Phase I clinical trials (CTR20223258, http://www.chinadrugtrials.org.cn/clinicaltrials.searchlist.dhtml, accessed on 19 December 2022), we have performed a parallel measurement on two commercially available APTT reagents (ellagic acid and colloidal silica APTT reagents). The purpose of this study is to evaluate their sensitivity in assessing the APTT prolongation of dHG-5 utilizing a semi-automated coagulometer. Moreover, we elucidated the underlying mechanism responsible for the observed discrepancy. In order to demonstrate the universality of sensitivity for FG compounds, dHLFG-4—another depolymerized FG from *Holothuria leucospilota* with a Mw of 5.20 kDa—was used for comparation. The results revealed that the sensitivity of dHG-5 and dHLFG-4 towards the ellagic acid APTT reagent was found to be twice that of the colloidal silica APTT reagent, which may be attributed to the comparatively weaker impact of the ellagic acid APTT reagent on the anti-iXase activity of dHG-5 and dHLFG-4 compared with the colloidal silica APTT reagent. 

## 2. Results

### 2.1. APTT Prolongation In Vitro

#### 2.1.1. Human Coagulation Control Plasma

**Figure 1:**
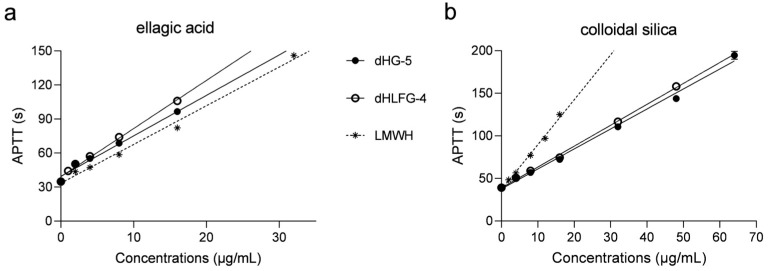
The APTT prolongation of compounds in human coagulation control plasma using ellagic acid (**a**) and colloidal silica (**b**) APTT reagents. All experiments were carried out in duplicate, and results were expressed as mean ± standard deviation (SD). (*n* = 2, mean ± SD).

The APTT prolongation induced by dHG-5 and dHLFG-4 was evaluated in human coagulation control plasma, using LMWH (enoxaparin) as a control (Figure 1 and Table 1). The results were consistent with our previous study [18]. They showed that the APTT prolongation of dHG-5 and dHLFG-4 was slightly more potent than that of LMWH when using the ellagic acid APTT reagent, since the concentrations required for doubling the baseline APTT value (EC_2.0×_) were 8.45 μg/mL, 7.17 μg/mL and 10.59 μg/mL, respectively. While the APTT prolongation of LMWH (with EC_2.0×_ value of 7.80 μg/mL) was more potent than that of dHG-5 and dHLFG-4 (with EC_2.0×_ values of 15.98 and 17.21 μg/mL, respectively) when using the colloidal silica APTT reagent. Comparative analysis revealed no significant disparities in the APTT prolongation of LMWH in human coagulation control plasma when employing colloidal silica (with EC_2.0×_ value of 7.80 μg/mL) and ellagic acid APTT reagents (with EC_2.0×_ value of 10.59 μg/mL). While the EC_2.0×_ of dHG-5 and dHLFG-4 using the colloidal silica APTT reagent was twice as high as that obtained with the ellagic acid APTT reagent. The results suggest that FG compounds exhibit a higher sensitivity to the ellagic acid APTT reagent in prolonging human plasma APTT, which is different from that of LMWH.

#### 2.1.2. Fresh Rat Plasma

**Figure 2:**
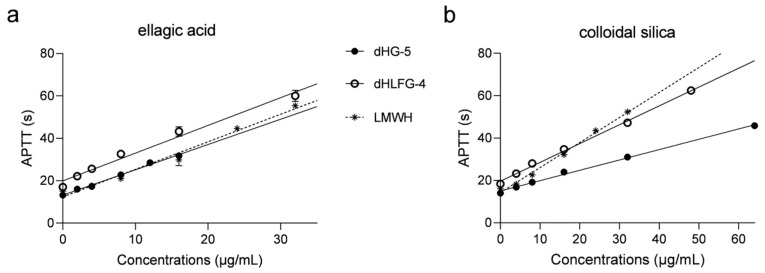
The APTT prolongation of compounds in rat plasma using ellagic acid (**a**) and colloidal silica (**b**) APTT reagents. (*n* = 2, mean ± SD).

The APTT prolongation of dHG-5 and dHLFG-4 in rat plasma was also evaluated, with results consistent with those obtained with human coagulation control plasma (Figure 2 and Table 2). The APTT prolongation of dHG-5 and dHLFG-4 (with EC_2.0×_ values of 11.06 and 10.83 μg/mL, respectively) was slightly more potent compared to LMWH (with EC_2.0×_ values of 13.70 μg/mL) when using the ellagic acid APTT reagent. While the potency of LMWH (with EC_2.0×_ value of 15.16 μg/mL) was found to be higher than that of dHG-5 and dHLFG-4 (with EC_2.0×_ values of 19.18 and 26.76 μg/mL, respectively) when using the colloidal silica APTT reagent. Comparative analysis of the data showed that the APTT prolongating efficacy of dHG-5 and dHLFG-4 when using the colloidal silica APTT reagent (with EC_2.0×_ values of 26.76 and 19.18 μg/mL, respectively) was approximately half as potent compared to using the ellagic acid APTT reagent (with EC_2.0×_ values of 11.06 and 10.83 μg/mL, respectively). While the APTT prolongation of LMWH in rat plasma showed no significant differences between using colloidal silica and ellagic acid APTT reagents (with EC_2.0×_ values of 13.70 and 15.16 μg/mL, respectively). These results support the conclusion that FG compounds are more sensitive to the ellagic acid APTT reagent than to the colloidal silica APTT reagent, regardless of whether it is tested in human or rat plasma.

### 2.2. APTT Prolongation In Vivo

**Figure 3:**
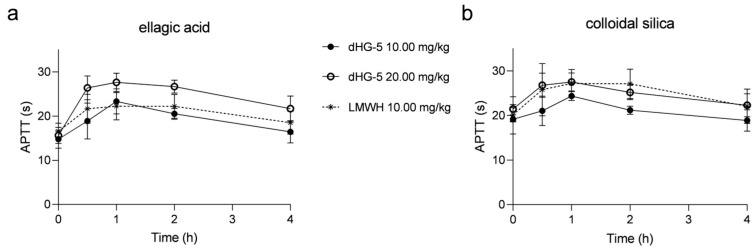
The prolongation of APTT at different time points in rats treated with dHG-5 and LMWH: APTT analysis using ellagic acid (**a**) and colloidal silica (**b**) APTT reagents. (*n* = 6–8, mean ± SD).

To investigate the consistency of colloidal silica and ellagic acid APTT reagents in evaluating the APTT prolongation of FG compounds in vivo and in vitro, we also assessed the APTT prolongation with dHG-5 and LMWH in rats. After subcutaneous administration to rats, the effects of dHG-5 and LMWH on rat plasma APTT was assessed at various time intervals. The prolongation of APTT with dHG-5 at a dose of 10 mg/kg was found to be comparable to that observed with LMWH when using the ellagic acid APTT reagent, although weaker than that observed with dHG-5 at a dose of 20 mg/kg. However, the prolongation of APTT with dHG-5 at a dose of 20 mg/kg was equivalent to that achieved with LMWH at a dose of 10 mg/kg when using the colloidal silica APTT reagent, which exhibited greater potency than dHG-5 at a dose of 10 mg/kg (Figure 3). The prolongation ratio of APTT in rats treated with dHG-5 and LMWH at different time points was compared between using ellagic acid and colloidal silica APTT reagents. (Table 3). The APTT ratio of dHG-5 at different time points was significantly higher when using the ellagic acid APTT reagent compared to the colloidal silica APTT reagent, regardless of the dosage of dHG-5 (10 or 20 mg/kg, Table 3, *p* < 0.05), while the APTT ratio of LMWH showed no significant differences when using colloidal silica and ellagic acid APTT reagents (Table 3, *p* > 0.05). The above results demonstrated that FG compounds exhibited a higher sensitivity to the ellagic acid APTT reagent compared to the colloidal silica APTT reagent in both in vitro and in vivo assessments of APTT prolongation. Conversely, no significant differences were observed for LMWH when either colloidal silica or ellagic acid APTT reagents were used.

### 2.3. Contact Activation and iXase Inhibition

**Figure 4:**
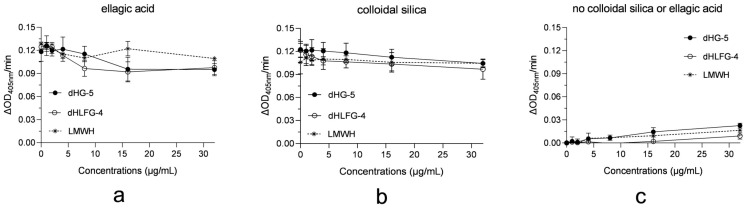
The impact of compounds on APTT reagents for activating human coagulation control plasma. The results were expressed as the rate of change of absorbance at 405 nm (ΔOD_405 nm/min_). The contact activation of the ellagic acid APTT reagent (**a**), and the colloidal silica APTT reagent (**b**) in human coagulation control plasma with compounds, and the contact activation of compounds without ellagic acid or colloidal silica APTT reagents (**c**). (*n* = 2, mean ± SD).

To clarify the factors related to the differences in APTT prolongation potency of dHG-5 and dHLFG-4 caused by colloidal silica and ellagic acid APTT reagents, we assessed the impact of dHG-5, dHLFG-4 and LMWH on these APTT reagents for activating human coagulation control plasma. The results demonstrated that dHG-5, dHLFG-4 and LMWH exhibited negligible activation and had minimal impact on the activation of ellagic acid and colloidal silica APTT reagents in human coagulation control plasma (Figure 4). This suggests that the impact of dHG-5 and dHLFG-4 on human plasma activation by APTT reagents may not be the primary determinant for the observed differences in APTT prolongation potency of dHG-5 and dHLFG-4 when using colloidal silica or ellagic acid APTT reagents. 

**Figure 5:**
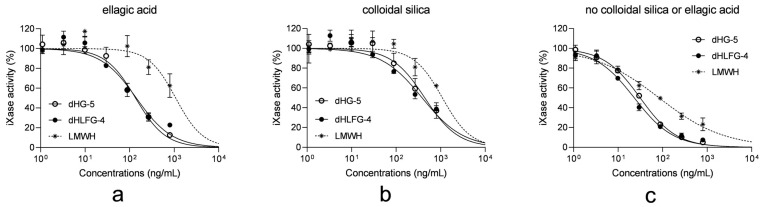
The impact of APTT reagents on the anti-iXase activity of compounds. The anti-iXase activity of dHG-5, dHLFG-4 and LMWH with the ellagic acid APTT reagent (**a**), the colloidal silica APTT reagent (**b**), and without ellagic acid or colloidal silica APTT reagents (**c**). (*n* = 2, mean ± SD).

Considering that the anticoagulant activities of dHG-5 and dHLFG-4 primarily arise from their anti-iXase activities, we investigated the impact of these APTT reagents on the anti-iXase activity of dHG-5 and dHLFG-4 [18]. The results showed that in the absence of ellagic acid or colloidal silica APTT reagents, the anti-iXase activity of dHG-5, dHLFG-4, and LMWH exhibited enhanced potency. This suggests that both the ellagic acid and colloidal silica APTT reagents attenuated their anti-iXase activity (Figure 5 and Table 4). However, the extent of this effect varied. In the presence of the ellagic acid APTT reagent, the anti-iXase potency of dHG-5 and dHLFG-4 exhibited an approximately twofold increase (with IC_50_ values of 136.00 ng/mL and 141.60 ng/mL, respectively) compared to that observed in the presence of the colloidal silica APTT reagent (with IC_50_ values of 440.00 and 383.40 ng/mL, respectively). While the anti-iXase potency of LMWH in the presence of the ellagic acid APTT reagent (with IC_50_ value of 1084.00 ng/mL) was found to be comparable to that observed with the colloidal silica APTT reagent (with an IC_50_ value of 1092.00 ng/mL). The above findings may provide an explanation for the heightened sensitivity of FG compounds to the ellagic acid APTT reagent in plasma APTT analysis.

## 3. Discussion

FGs isolated from sea cucumbers have been reported to exhibit potent iXase inhibitory, anticoagulant, and antithrombotic activities [11,17,18]. Plasma APTT analysis as a routine screening test plays a crucial role in the discovery and development of novel anticoagulants [19,20,27]. In this study, we observed that two types of APTT reagent exhibit variations in their suitability for assessing the anticoagulant effect of FG compounds, which is different from LMWH. The sensitivity of FG compounds to the ellagic acid APTT reagent in assessing their APTT prolongation is higher compared to the colloidal silica APTT reagent, both in vitro and in vivo. The underlying cause was investigated by examining the effect of APTT reagent on the anti-iXase activity exhibited by these compounds, as well as evaluating the impact of these compounds on the activation of the human plasma contact activation pathway triggered by APTT reagent. The results demonstrated that FG compounds and LMWH had no discernible effect on the activation of ellagic acid and colloidal silica APTT reagents in human coagulation control plasma. Conversely, ellagic acid and colloidal silica APTT reagents attenuated the anti-iXase activity of FG compounds and LMWH. These results suggest that FG compounds and LMWH may exhibit enhanced in vivo anticoagulation potency compared to their in vitro effects as assessed by the APTT method. Comparative analysis revealed that the inhibitory potency of ellagic acid and colloidal silica APTT reagents on the anti-iXase activity of LMWH is almost the same. While the inhibitory effect of the ellagic acid APTT reagent on the anti-iXase activity of FG compounds is weakened by approximately twofold in comparison to its effect in the presence of the colloidal silica APTT reagent. This confirms that the higher sensitivity of FG compounds to the ellagic acid APTT reagent in plasma APTT analysis is due to the lower inhibition of the ellagic acid APTT reagent on the anti-iXase activity of FG compounds compared to the colloidal silica APTT reagent, because the anticoagulant activities of dHG-5 and dHLFG-4 primarily arise from their anti-iXase activities [18].

APTT is most widely used to evaluate the contact activation pathway and the common pathway of coagulation, including abnormalities of related coagulation factors, lupus anticoagulant screening tests, and inhibitors targeting the contact activation pathway. However, it has been reported that various commercially available APTT reagents exhibited different sensitivities to coagulation factor abnormalities and anticoagulant activities’ evaluation of drugs [26,31,32]. The responsiveness of APTT reagents varies depending on the composition, mainly including the source and concentration of phospholipids and the type of surface activator [33,34]. It has been reported that colloidal silica impaired the activation of factor IX, and that different APTT reagents composed of various phospholipids and activators showed variability in evaluating the activity of factor VIII and factor IX, which might be associated with the attenuated iXase activity of FG compounds when using the colloidal silica APTT reagent [35,36]. Meanwhile, ellagic acid and colloidal silica APTT reagents showed the same degrees of effect on the anti-iXase activity of LMWH in this study, which might indicate that the weaker sensitivity of FG compounds to the colloidal silica APTT reagent was related to their properties, or that it was merely due to the weaker anti-iXase activity of LMWH compared with its activities of anti-factor Xa and IIa [37,38]. However, due to the heterogeneous composition of these reagents, we could not elucidate the precise underlying mechanism responsible for the attenuation of anti-iXase activity by ellagic acid and colloidal silica APTT reagents. 

This study found the higher sensitivity of the ellagic acid APTT reagent in assessing the anticoagulant activities of FG compounds, and preliminarily revealed the underlying cause. It highlights the importance of assessing the suitability of different commercially available APTT reagents to ensure a comprehensive evaluation of anticoagulant activity, thereby minimizing the risk of overlooking potential novel and effective anticoagulant candidates, particularly for inhibitors targeting the contact activation pathway and FG compounds derived from sea cucumbers. 

## 4. Materials and Methods

The anticoagulant candidate dHG-5 was provided by Jiuzhitang Co., Ltd. (Beijing, China), and was prepared using the β-eliminative depolymerization method from natural FG extracted from dried sea cucumbers (*Holothuria fuscopunctata*). According to our previous study, native FG had a backbone consisting of repeated {4)-_D_-glucuronic acid (GlcA)-β(1,3)-*N*-acetyl-*_D_*-galactosamine (GalNAc)-β(1,} disaccharide units, and abundant α-*_L_*-fucose sulfate (FucS) branches linked to C3 of each GlcA residue in the backbone. The types of FucS branches in the native FG were *_L_*-fucose-3,4-disulfates (Fuc_3S4S_, 85%), *_L_*-fucose-2,4-disulfates (Fuc_2S4S_, 10%), and *_L_*-fucose-4-sulfates (Fuc_4S_, 5%). The molar percentages of the main oligosaccharides with degrees of polymerization of 5, 8, 11, 14, 17, 20, 23, 26, 29, and >29 in dHG-5 were estimated as 4.86, 17.39, 17.55, 15.93, 13.62, 11.22, 6.62, 4.74, 2.95, and 5.12%, respectively, according to the proportion of the peak area in HPGPC [17,18]. The depolymerized FG, dHLFG-4, was obtained using the peroxidative depolymerization method from dried sea cucumbers (*Holothuria leucospilota*) purchased from local markets in Qionghai, Hainan Province, China (unpublished data). LMWH (Enoxaparin, 0.4 mL 4000 AXaIU, Mw~4.5 kDa) was purchased from Sanofi-Aventis (Paris, France). The ellagic acid APTT reagent was purchased from BJ MDC (Beijing, China) and the colloidal silica APTT reagent was purchased from HemosIL (Tokyo, Japan). The detailed composition can be found in Table 5. The human coagulation control plasma was purchased from Teco Medical (Niederbayern, Germany). Biophen factor VIII: C kit and kallikrein chromogenic substrate CS-31(02) were purchased from Hyphen BioMed (Paris, France). Recombinant coagulation factor VIII was purchased from Bayer HealthCare LLC (Berkeley, CA, USA). All other chemical reagents were commercially available and of analytical grade. 

Sprague-Dawley rats (male, 220–250 g, License No. SCXK 2019-0004) were purchased from Hunan SJA Laboratory Animal Co., Ltd. (Hunan, China). The rats were kept for 7 days in a specific pathogen-free environment with adequate water and food, thermoregulatory conditions (22–25 °C), and a 12-h light–dark cycle. In this study, all animals were raised and manipulated following protocols approved by the Animal Experimentation Ethics Committee of the South-Central Minzu University, Wuhan, China (approval number: 2020-SCUEC-013).

### 4.1. Plasma APTT Analysis

The APTT prolongation of compounds in human and rat plasma was determined with a coagulometer (TECO MC-2000, Niederbayern, Germany) using APTT reagent as previously described [18]. Briefly, a 5 μL sample dissolved in Tris/HCl buffer (0.02 M, pH 7.4) and 45 μL plasma were pipetted into a cuvette, followed by incubation at 37 °C for 2 min. Subsequently, 50 μL of APTT reagent was added and incubated for an additional 3 min. Finally, 50 μL of CaCl_2_ reagent was introduced, and the clotting time was recorded. The assays were conducted in duplicate. The compound concentrations in plasma and clotting times were fitted linearly using GraphPad Prism 8.0 software (GraphPad Software, San Diego, CA, USA).

### 4.2. APTT Prolongation In Vivo

The rats were randomly assigned to groups based on their body weight, with 6 rats allocated to the dHG-5 10.00 mg/kg group, 8 rats allocated to the dHG-5 20.00 mg/kg group, and 7 rats allocated to the LMWH 10.00 mg/kg group. The compounds were dissolved in 0.9% sodium chloride injection and administered subcutaneously to rats following randomization. The venous blood samples (approximately 0.4 mL), anticoagulated with 3.8% sodium citrate, were collected from the rats’ orbital venous plexus before and after administration of the compounds at 0.5-h, 1-h, 2-h, and 4-h time points. The samples were then promptly centrifuged at 3000× *g* for 10 min, followed by ex vivo analysis of plasma sample APTT using the method described above.

### 4.3. Contact Activation in Plasma

The activation effects of ellagic acid and colloidal silica APTT reagents in plasma were assessed through chromogenic substrate hydrolysis assays as previously described with minor modifications [18]. A sample of 30 μL diluted plasma (1:3, plasma: Tris/HCl buffer (*v*/*v*)) and 30 μL APTT reagent with or without compounds (1:1, APTT reagent: compounds dissolved in Tris/HCl buffer (*v*/*v*)) were mixed in 96-well plates and incubated at 37 °C for 1 min. An additional 30 μL of 0.3 mM CS-31(02) was then added. The contact activation in plasma exhibited a positive correlation with the release rate of p-nitroaniline from CS-31(02), which was monitored at 405 nm on a microplate reader every 15 s for 2 min (kinetic method). The results were expressed as the rate of change of absorbance at 405 nm (ΔOD_405 nm/min_). The experiments were independently replicated in duplicate.

### 4.4. Inhibition for iXase 

The anti-iXase activity of the compounds was assessed using Biophen factor VIII:C kits (containing R1, R2, and R3 solutions) and measured with a Victor Nivo Microplate Reader (PerkinElmer, Waltham, MA, USA) according to previously established protocols [18]. Briefly, 30 μL of solution containing compounds with or without APTT reagents (1:1, compounds dissolved in Tris/HCl buffer: APTT reagent (*v*/*v*)), 30 μL factor VIII (2 IU/mL), and 30 μL R2 solution (containing 60 nM factor IXa, human thrombin, phosphatidylcholine/phosphatidylserine and Ca^2+^) were mixed in 96-well plates and incubated at 37 °C for 2 min. Then, 30 μL R1 solution (containing 50 nM factor X and thrombin inhibitor) was added. After incubation for 1 min at 37 °C, the residual factor Xa activity was measured by adding 30 μL R3 solution (factor Xa chromogenic substrate SXa-11). The iXase activity exhibited a positive correlation with the release rate of p-nitroaniline from SXa-11, which was monitored at 405 nm every 15 s for 2 min on a microplate reader (kinetic method). Using the ΔOD_405 nm/min_ in the absence of compounds as 100%, the relative activity of compounds was calculated. The concentrations of the compounds were plotted against their relative activities and fitted using the GraphPad Prism 8.0 software (GraphPad Software, San Diego, CA, USA) with a model of log(inhibitor) vs. response-Variable slope (four parameters). The IC_50_ values of the compounds were calculated and used to compare the potency of the activity. The experiments were independently replicated in duplicate.

### 4.5. Statistical Analysis 

Statistical analysis was conducted using the GraphPad Prism v8.0.2 software (GraphPad Software, San Diego, CA, USA). The data were presented as the mean ± SD. The normality of the data was tested through the Shapiro–Wilk test. A two-way one-way analysis of variance (ANOVA) followed by Sidak’s multiple comparison test was used for determining mean differences. Statistical significance was defined as *p* value ≤ 0.05. 

## 5. Conclusions

Considering that various commercially available APTT reagents varied in sensitivity for evaluating the anticoagulant activities of drugs, such as heparin, dabigatran, and rivaroxaban, the sensitivity of ellagic acid and colloidal silica APTT reagents—two commonly used APTT reagents in routine laboratory anticoagulant screening tests for evaluating anticoagulant activities of two depolymerized FGs derived from sea cucumbers—were comparatively assessed in this paper. 

As potent iXase inhibitors, two depolymerized FGs, dHG-5 and dHLFG-4, both exhibited effective APTT prolongation both in vitro and in vivo, especially when using ellagic acid APTT reagents. Given that FG compounds had no discernible effect on the activation of ellagic acid and colloidal silica APTT reagents in human coagulation control plasma, the attenuated anti-iXase activities of FG compounds by ellagic acid and colloidal silica APTT reagents were confirmed, especially when using colloidal silica APTT reagents. Combined with the results of previous studies showing that the anticoagulant activities of FG compounds primarily arise from their anti-iXase activities, it can be concluded that the sensitivity of FG compounds to the ellagic acid APTT reagent is higher compared to the colloidal silica APTT reagent in assessing their anticoagulant activities, both in vitro and in vivo, which attributes to the weaker inhibition of the ellagic acid APTT reagent on the anti-iXase activity of FG compounds. In addition, this study could provide scientific evidence for the selection of APTT reagents in the evaluation of the anticoagulant activity of FGs.

## Figures and Tables

**Table 1:** APTT prolongating potency of compounds in human coagulation control plasma using ellagic acid and colloidal silica APTT reagents. (*n* = 2, mean ± SD).

Compounds	EC_2.0×_ (APTT, μg/mL) ^1^	
	Ellagic Acid	Colloidal Silica
dHG-5	8.45 ± 0.50	17.21 ± 1.20
dHLFG-4	7.17 ± 0.41	15.98 ± 0.55
LMWH	10.59 ± 0.68	7.80 ± 0.33

^1^ Compound concentration required for doubling baseline APTT value.

**Table 2:** APTT prolongating potency of compounds in rat plasma using ellagic acid and colloidal silica APTT reagents. (*n* = 2, mean ± SD).

Compounds	EC_2.0×_ (APTT, μg/mL) ^1^	
	Ellagic Acid	Colloidal Silica
dHG-5	11.06 ± 0.47	26.76 ± 1.11
dHLFG-4	10.83 ± 1.19	19.18 ± 0.75
LMWH	13.70 ± 1.21	15.16 ± 0.66

^1^ Compound concentration required for doubling baseline APTT value.

**Table 3:** The APTT ratio at different time points in rats treated with dHG-5 and LMWH. (*n* = 6–8, mean ± SD).

Compounds	Doses (mg/kg)	APTT Ratio ^1^									
		Ellagic Acid					Colloidal Silica				
		0 h	0.5 h	1 h	2 h	4 h	0 h	0.5 h	1 h	2 h	4 h
dHG-5	10.00	1.00 ± 0.14 ^#^	1.28 ± 0.20 ^#^	1.58 ± 0.20 *	1.39 ± 0.08 *	1.1 ± 0.17 ^#^	1.00 ± 0.17	1.10 ± 0.17	1.27 ± 0.05	1.11 ± 0.05	0.99 ± 0.13
dHG-5	20.00	1.00 ± 0.13 ^#^	1.64 ± 0.18 *	1.74 ± 0.14 *	1.68 ± 0.10 *	1.36 ± 0.19 *	1.00 ± 0.13	1.25 ± 0.13	1.28 ± 0.09	1.17 ± 0.08	1.04 ± 0.12
LMWH	10.00	1.00 ± 0.11 ^#^	1.24 ± 0.20 ^#^	1.34 ± 0.21 ^#^	1.34 ± 0.16 ^#^	1.12 ± 0.14 ^#^	1.00 ± 0.08	1.27 ± 0.29	1.34 ± 0.15	1.33 ± 0.16	1.09 ± 0.19

^1^ APTT ratio = APTT values at different time points/APTT values at 0 h. * *p* < 0.05, ^#^ *p* > 0.05, ellagic acid vs. colloidal silica.

**Table 4:** The anti-iXase activity of compounds with and without APTT reagents. (*n* = 2, mean ± SD).

Compounds	IC_50_ of Anti-iXase (ng/mL) ^1^		
	Ellagic Acid	Colloidal Silica	No Colloidal Silica or Ellagic Acid
dHG-5	136.00 ± 16.07	440.00 ± 64.39	31.28 ± 1.54
dHLFG-4	141.60 ± 21.54	383.40 ± 65.65	23.09 ± 1.83
LMWH	1084.00 ± 423.40	1092.00 ± 272.67	83.19 ± 7.67

^1^ The compound concentration required for inhibiting 50% iXase activity.

**Table 5:** Activators and phospholipids in each APTT reagent.

Reagent	Manufacturer	Phospholipid	Activator
APTT reagent kit	BJ MDC	Cephalin obtained from rabbit brain	Ellagic acid
SynthASil kit	HemosIL	Synthetic phospholipid	Colloidal silica

## Data Availability

All data generated or analyzed during this study are included in this published article.
